# Following patient pathways to psycho‐oncological treatment: Identification of treatment needs by clinical staff and electronic screening

**DOI:** 10.1002/pon.4675

**Published:** 2018-03-24

**Authors:** Fanny L. Loth, Verena Meraner, Bernhard Holzner, Susanne Singer, Irene Virgolini, Eva M. Gamper

**Affiliations:** ^1^ Department of Psychiatry, Psychotherapy and Psychosomatics Medical University of Innsbruck Innsbruck Austria; ^2^ Faculty of Psychology and Sport Science University of Innsbruck Innsbruck Austria; ^3^ Institute of Medical Biostatistics, Epidemiology and Informatics, University Medical Centre Johannes Gutenberg University Mainz Mainz Germany; ^4^ University Cancer Centre Mainz Mainz Germany; ^5^ Department for Nuclear Medicine Medical University of Innsbruck Innsbruck Austria; ^6^ Innsbruck Institute of Patient‐centered Outcome Research Innsbruck Austria

**Keywords:** cancer, oncology, psychosocial distress, quality of care, screening

## Abstract

**Objective:**

In this retrospective investigation of patient pathways to psycho‐oncological treatment (POT), we compared the number of POT referrals before and after implementation of electronic screening for POT needs and investigated psychosocial predictors for POT wish at a nuclear medicine department.

**Methods:**

We extracted medical chart information about number of referrals and extent of follow‐up contacts. During standard referral (November 2014 to October 2015), POT needs were identified by clinical staff only. In the screening‐assisted referral period (November 2015 to October 2016), identification was supported by electronic screening for POT needs. Psychosocial predictors for POT wish were examined using logistic regression.

**Results:**

We analysed data from 487 patients during standard referral (mean age 56.4 years; 60.2% female, 88.7% thyroid carcinoma or neuroendocrine tumours) of which 28 patients (5.7%) were referred for POT. Of 502 patients in the screening‐assisted referral period (mean age 57.0 years; 55.8% female, 86.6% thyroid carcinoma or neuroendocrine tumours), 69 (13.7%) were referred for POT. Of these, 36 were identified by psycho‐oncological (PO) screening and 33 by clinical staff. After PO‐screening implementation, referrals increased by a factor of 2.4. The strongest predictor of POT wish was depressive mood (P < .001). During both referral periods, about 15% of patients visited the PO outpatient unit additionally to inpatient PO consultations.

**Conclusions:**

Our results provide evidence from a real‐life setting that PO screening can foster POT referrals, reduce barriers to express the POT wish, and hence help to meet psychosocial needs of this specific patient group. Differences between patients' needs, wish, and POT uptake should be further investigated.

## 1 BACKGROUND

Patients with cancer with high psychosocial distress are less likely to adhere to treatment recommendations,^1^ show poorer satisfaction with care,^2^ adapt less to living with cancer,^3^ are more vulnerable to disability,^4^, ^5^ and have poorer quality of life (QOL).^6^ Providing patients with cancer with adequate psychosocial support and treatment reduces distress and improves QOL.^6^, ^7^, ^8^ It also has a positive effect on cost‐utility ratios and accounts for only 3% of total health care costs.^9^

Consequently, adequate psycho‐oncological (PO) care is becoming a standard practice,^10^, ^11^ and it is a certification criterion for cancer centres (OnkoZert) in Austria and Germany.^12^ Still, an alarmingly high percentage of distressed patients is unrecognised and untreated in clinical practice.^13^, ^14^, ^15^, ^16^, ^17^ This is particularly true for patients with thyroid carcinoma (ThyCa) who report to be considered to have “the good cancer,” which not only imposes additional burden but also impedes their access to psychosocial care.^18^, ^19^ These patients' unrecognised QOL impairments and unmet psychosocial treatment needs have only recently gained attention.^17^, ^20^, ^21^

Generally, few patients self‐refer to PO counselling,^17^, ^22^, ^23^ and health care professionals (HCPs) often fail to identify patients needing psycho‐oncological treatment (POT) for reasons such as time constraints, lack of human resources, focus on physical aspects, or difficulties in recognising and addressing emotional problems.^10^, ^12^, ^20^ To overcome this problem, it is essential to adequately identify and measure distress in clinical routines,^24^ eg, through stepped‐care approaches, such as questionnaire‐assisted screening followed by personal triage.^5^, ^25^, ^26^, ^27^ In such approaches, it is essential that patients are screened regularly (especially at times of higher risk), screening tools are as comprehensible and as brief as possible, and screening results should be immediately available. The use of electronic screening facilitates data collection, and questionnaire results are scored automatically and easily interpretable.^25^, ^26^, ^27^, ^28^ Although distress in patients with cancer is well investigated and promising screening measures have been developed,^5^, ^13^, ^17^, ^26^, ^29^, ^30^ little is known about how distressed patients are identified and referred for POT in real world outside a study setting.

We report on an electronic routine screening for QOL impairments and POT needs in a nuclear medicine department. Our main aim was to investigate pathways to POT before and after the implementation of electronic PO screening. We retrospectively analysed data addressing the following aims:

Aim 1 Investigation of pathways to POT during a standard referral period and a screening‐assisted referral period
Aim 2 Investigation of psychosocial predictors for POT wish
Aim 3 Investigation of QOL differences in patients referred by HCPs and via screening

Patients identified by PO‐screening show lower scores on emotional functioning (EF) and role functioning (RF) scales than patients identified by HCPs.

## 2 METHODS

### 2.1 Patients and data sets

#### 2.1.1 Clinical setting and data collection procedure

The 2 largest diagnostic groups at the Department of Nuclear Medicine at the Medical University of Innsbruck are patients with ThyCa (curative approach) and patients with neuroendocrine tumour (palliative approach). Patients are usually admitted for inpatient stay to receive therapy or to undergo examinations involving radiopharmaceuticals.

In 2011, the department implemented a routine electronic QOL‐monitoring system to capture symptoms and other QOL‐related issues that may be relevant to patients.^31^ Specialised software (the Computer‐based Health Evaluation System)^32^ permits the electronic collection, calculation, and interpretation of patient‐reported outcome data with immediate access to the results.

At each inpatient stay, patients complete the QLQ‐C30^33^ (plus a disease‐specific module) using a tablet PC. Patients with ThyCa complete the assessment up to 2 times a year, and patients with neuroendocrine tumour up to 3 times. A screening tool to identify patients with POT needs was included in the routine monitoring in November 2015. Patients may decline to participate or to skip individual questions without giving reasons. Routine‐monitoring results are available for HCPs via all ward computers. For the present retrospective data analyses, ethical approval was not required according to the ethical review committee of the Medical University of Innsbruck.

#### 2.1.2 Procedures for referral to the hospital's PO unit

Standard POT referral (before November 2015): The standard procedure for referral to the hospital's PO unit (hereafter termed standard referral) was via an HCP who approaches the patient and, with the patient's consent, initiates POT referral.
PO‐screening referral (since November 2015): The implementation of PO screening (described in detail below) included a second referral branch. Results of the PO screening were immediately available to HCPs and to the PO service. If a patient expressed an explicit POT wish, a psycho‐oncologist approached them directly.

#### 2.1.3 Patient sample and data extraction

Patients were eligible for PO screening if diagnosed with cancer; older than 18 years; PO naïve (ie, no previous referrals for POT in the hospital); and did not have brain metastases, a diagnosis of dementia, or other cognitive impairments.

Sociodemographic and clinical data, including information on referrals to POT and number and extent of PO consultations, were gathered from hospital charts.

For the investigation of our aims, we extracted data from the following periods:

For aims 1 and 2, we extracted data from the standard referral period November 2014 to October 2015, in which routine QOL‐monitoring with the QLQ‐C30 was paused, and a screening‐assisted referral period, November 2015 to October 2016, in which in addition to the PO screening, the QOL monitoring with the QLQ‐C30 was started again.

For aim 3, we used data from the same screening‐assisted referral period and for comparison extracted data from a standard referral period in which QOL monitoring with the QLQ‐C30 had been performed (November 2013 to October 2014).

### 2.2 Assessment instruments

#### 2.2.1 EORTC QLQ‐C30

This internationally validated 30‐item questionnaire assesses cancer‐specific QOL.^33^ It comprises 5 functional scales (physical, social, role, cognitive, and emotional functioning); 9 symptom scales (fatigue, nausea/vomiting, pain, dyspnoea, sleep disturbances, appetite loss, constipation, diarrhoea, and financial impact); and a global QOL scale. Linear‐converted scale scores range from 0 to 100. High functional scale scores and global health status/QOL scores indicate better functioning and high symptom scale scores represent higher symptom burden.

#### 2.2.2 PO‐screening tool

The screening tool to identify POT needs was constructed by collaboration between the hospital's PO unit and the nuclear medicine department. The tool was based on existing screenings (the Hornheide screening instrument [HSI] short form^34^ and the screening tool for POT in patients with breast cancer^29^) and adapted to local requirements. The adaptations included the exclusion of information on overall health and emotional well‐being as such questions already asked within the QLQ‐C30 and the inclusion of specific questions relevant for the clinical setting (eg, anxiety attacks). The final screening tool comprised 3 modules with a total of 11 questions*:

Module 1 Psychosocial distress and psychological/psychiatric pretreatment:
a HSI: Is there anything that causes emotional burden on you that is not related to the disease?
b HSI: Is there anybody you can talk to about your worries and fears?
c HSI: Is there anybody in your family particularly burdened because of your hospital stay?
d HSI: Are you able to calm down during the day?
e HSI: How well informed do you feel about your disease and the treatment?
f POT: Do you or did you ever suffer from a significantly depressive mood, occurring almost daily over a period of at least two weeks?
g POT: Are you or have you ever been in psychologic/psychotherapeutic/psychiatric treatment or care?
Module 2 Anxiety: As patients treated with radiopharmaceuticals must be isolated for several days, we included two questions with a special focus on anxiety attacks that could be answered with either yes or no: “Do you/did you ever suffer from anxiety attacks, in which you felt a sudden intense fear, trepidation, or unrest?” If answered affirmatively, “Are you concerned that you might experience such feelings during treatment?”
Module 3 POT wish was assessed using the statement: “We would like to give you the opportunity to talk to a psycho‐oncologist during your treatment at our department. Please inform us if you wish to do so” followed by “I would like to talk to a psycho‐oncologist” (yes/no).

The software generated a critical PO flag indicating potential treatment need if the cut‐off of 4 points was reached in module 1 (ie, min. 2 psychosocial problem areas identified), patients reported on history of anxiety attacks and fear of experiencing anxiety attacks during isolation in module 2, or expressed an explicit whish for POT (module 3).

### 2.3 Statistical analyses

Sample characteristics are shown as frequencies, means, standard deviations, and ranges.

Aim 1 To describe patient pathways to POT in the 2 different referral periods, we used absolute and relative frequencies.
Aim 2 For the investigation of psychosocial predictors of POT wish, we used binary logistic regression analyses with POT wish (yes/no) as the dependent variable. We included as potential predictors the questions from modules 1 and 2 of the PO‐screening tool and, based on findings from previous research^29^ QLQ‐C30 Emotional Functioning and Role Functioning scores as well as age and sex in a backward‐elimination regression.
Aim 3 Analyses of QLQ‐C30 score differences between patients with POT wish and patients referred by HCPs were conducted using Student *t* test.

## 3 RESULTS

### 3.1 Aim 1: investigation of PO care pathways in the 2 referral periods

#### 3.1.1 Sample characteristics

We extracted sociodemographic and clinical data for all 487 PO‐naïve patients admitted to the department in the standard referral period (60.2% female, age 56.4 ± 15.8 years) and from 502 PO‐naïve patients in the screening‐assisted period (55.8% female, age 57.0 ± 15.9 years). Of the latter, 286 patients (57.0%) were assessed using the PO‐screening tool (62.2% female, age 53.8 ± 14.9 years). Further details are given in Table 1.

**Table 1:** Sample characteristics

	Aim 1 and Aim 2			Aim 3		
	Standard Referral 2014/2015	Screening‐Assisted Referral 2015/2016		Patients with Potential POT Need		
	Total (N = 487)	Total (N = 502)	Included in PO Screening (N = 286)	HCP Identified 2013/2014 (N = 29)	HCP Identified 2013/2014 (N = 18)^a^	PO‐Screening Identified 2015/2016 (N = 41)^b^
Sex N (%)						
Female	194 (39.8)	280 (55.8)	178 (62.2)	26 (89.7)	17 (94.4)	30 (73.2)
Male	293 (60.2)	222 (44.2)	108 (37.8)	3 (10.3)	1 (5.6)	11 (26.8)
Age mean (SD)	56.4 (15.8)	57.0 (15.9)	53.8 (14.9)	48.4 (14.6)	49.2 (14.5)	55.0 (14.9)
Diagnosis N (%)						
ThyCa	326 (66.9)	344 (68.5)	223 (78.0)	15 (51.7)	12 (66.7)	32 (78.0)
NETs	106 (21.8)	91 (18.1)	52 (18.2)	7 (24.1)	4 (22.2)	7 (17.1)
Other	55 (11.3)	67 (13.4)	11 (3.8)	7 (24.1)	2 (11.1)	2 (4.9)

Abbreviations: HCP, health care professional; NET, neuroendocrine tumour; PO, psycho‐oncological; POT, psycho‐oncological treatment.

Only valid percentages are reported.

a Only patients with QLQ‐C30 data for the time of referral to POT.

b Only patients with positive PO‐screening and QLQ‐C30 data.

#### 3.1.2 Referrals for POT from standard referral vs screening‐assisted referral

In the standard referral period, 28 patients were successfully referred to POT (ie, 5.7% of PO‐naïve patients admitted to the department in this period were seen by a psycho‐oncologist).

**Figure 1:**
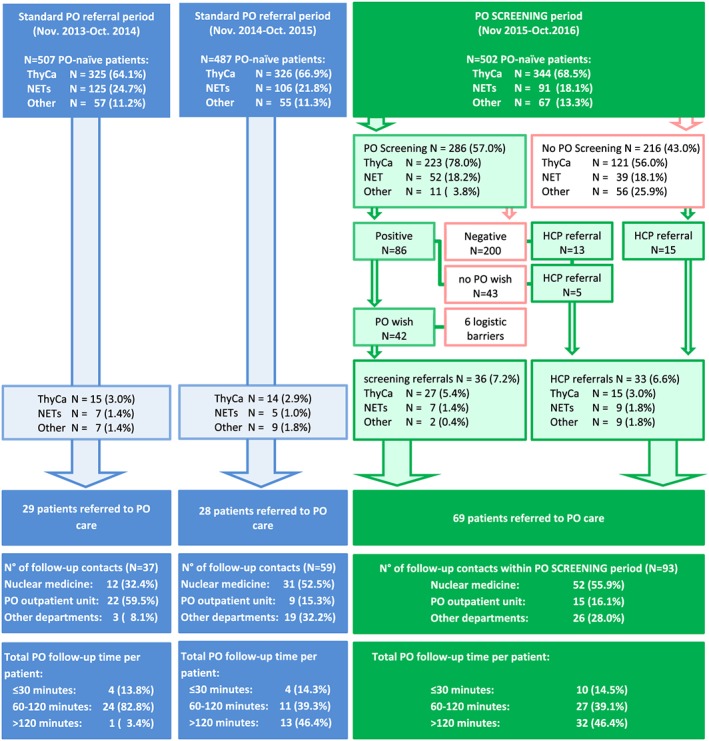
Patient PO care pathways. HCP, health care professional; NET, neuroendocrine tumour; PO, psycho‐oncological; ThyCa, thyroid carcinoma

During the screening‐assisted period, a total of 114 patients (22.7% of all PO‐naïve patients admitted to the department) with potential POT needs were identified. Of these, 86 patients were identified by the PO screening (81 without such reporting emotional burden that is not related to the disease), but only 42 of these expressed an explicit POT wish and 43 did not (1 patient did not answer the question on POT wish). Six patients with POT wish could not be approached for administrative reasons. Twenty‐eight patients were referred by HCPs. Fifteen patients had not participated in PO screening. Thirteen had participated in screening but had not exceeded the cut‐off and had no POT wish. Five of these agreed to POT after being approached by an HCP. In summary, 69 patients (13.7% of PO‐naïve patients admitted to the department) were successfully referred for POT. Figure 1 shows a flow diagram of PO care pathways from November 2013 to October 2016.

#### 3.1.3 Extent of PO consultations in standard referral vs screening‐assisted referral

During the standard referral period, 67.9% (19 out of 28) had subsequent contacts after the first consultation. During the screening‐assisted referral period, this proportion was 58.0% (40 out of 69). Of all subsequent PO consultations after initial referral within the standard referral period, 31 contacts (52.5%) were at the Department of Nuclear Medicine, 9 (15.3%) at the PO outpatient unit, and 19 (32.2%) at other departments. Most of the patients' subsequent PO consultations during the screening‐assisted period likewise were at the Department of Nuclear Medicine (N = 52; 55.9%). Fifteen patients (16.1%) visited the PO outpatient unit, and 26 patients (28.0%) were followed‐up at another hospital department.

### 3.2 Aim 2: identification of psychosocial distress factors associated with POT wish

In the screening‐assisted referral period, 86 (30.1% of the 286 patients being screened) exceeded the cut‐off for potential POT needs. The most frequent issue reported by patients was a history of or current anxiety attacks. Table 2 shows separate frequencies of psychosocial issues reported in the screening for patients above and below the cut‐off.

**Table 2:** Psychosocial issues in patients with or without potential treatment needs

	Positive PO Screening			Nonpositive PO Screening		
	Yes	No		Yes	No	
Emotional burden not in relation to disease	40 (46.5)	46 (53.5)		17 (8.6)	180 (91.4)	
Lack of social support	10 (8.6)	76 (88.4)		6 (3.0)	192 (97.0)	
Burden on family through hospital stay	32 (38.1)	52 (61.9)		31 (15.7)	166 (84.3)	
Inability to calm down during the day	26 (30.6)	59 (69.4)		3 (1.5)	195 (98.5)
History of or current anxiety attacks	43 (50.0)	43 (50.0)		13 (6.6)	184 (93.4)	
Fear of anxiety attacks during isolation^a^	8 (19.0)	34 (81.0)		…	13 (100.0)	
History of or current depressive mood	31 (36.0)	55 (64.0)		9 (4.5)	189 (95.5)	
Previous or current psychiatric/or psychotherapeutic treatment	31 (36.0)	55 (64.0)		21 (10.6)	177 (89.4)	
	Good	Moderate	Poor	Good	Moderate	Poor
Level of information	64 (74.4)	21 (24.4)	1 (1.2)	175 (88.4)	22 (11.1)	1 (0.5)
	Yes	No		Yes	No	
POT wish	39 (45.9)	46 (54.1)		…	198 (100)	

Abbreviations: PO, psycho‐oncological; POT, psycho‐oncological treatment.

Only valid percentages are reported.

a n = 55 patients.

Logistic regression modelling resulted in a single predictor for POT wish: current or previous depressive mood. The odds for POT wish were more than 5 times higher in patients who had experienced depressive mood compared with those who had not (*P* < .001; OR = 5.7; 95% CI, 2.6‐12.3). A total of 66.1% of the patients expressing POT wish and having a history of or current depressive mood reported to be or to have been in psychotherapeutic or psychiatric treatment or care. Patients with and without former psychosocial care uptake differed significantly (*P* = .002) regarding previous or current depressive mood.

### 3.3 Aim 3: differences in QLQ‐C30 scores between patients identified by standard referral vs PO screening‐assisted referral

For the HCP referred subsample 2013/2014, we extracted data of 18 PO‐naïve patients with corresponding QLQ‐C30 results to the referral for POT care. For the comparing subsample of patients being identified as distressed by the PO‐screening, we were able to extract data from 41 PO‐naïve patients with available QLQ‐C30 data. The mean age of HCP referred patients was 49.2 ± 14.5 years, and 94.4% were female. Of the 41 patients with a positive PO‐screening, the mean age was 55.0 ± 14.9 years, and 73.2% were female. Details are given in Table 1.

On the basis of Meraner et al,^29^ we expected patients with a positive PO screening to show lower Emotional Functioning and Role Functioning scores. However, Emotional Functioning and Role Functioning scores did not significantly differ between patients identified by HCPs and patients identified by PO screening (*P* values .254, 95% CI, −24.3 to 6.6, and .277; 95% CI, −8.5 to 29.1) (Table 3). Similarly, no significant score differences were found for the remaining QOL scales.

**Table 3:** QOL differences in distressed patients identified by HCPs or via PO screening

	Referral Period 2013/2014 (N = 18)		Referral Period 2015/2016 (N = 41)				
	Mean	SD	Mean	SD	Mean Difference	95% CI	*P* Value
Physical functioning	80.0	21.3	77.7	22.1	2.3	−10.1 to 14.7	.714
Role functioning	71.3	28.5	61.0	35.1	10.3	−8.5 to 29.1	.277
Social functioning	65.7	27.1	63.0	34.9	2.7	−15.8 to 21.3	.769
Emotional functioning	47.2	24.4	56.1	28.4	−8.9	−24.3 to 6.6	.254
Cognitive functioning	60.2	33.4	61.0	30.2	−0.8	−18.4 to 16.9	.929
Global quality of life	55.6	21.5	56.9	24.0	−1.4	−14.1 to 11.4	.833
Fatigue	48.8	28.3	55.0	30.5	−6.2	−23.2 to 10.7	.463
Nausea/vomiting	11.1	17.1	10.2	15.3	0.9	−8.0 to 9.9	.833
Pain	23.1	31.4	27.6	31.8	−4.5	−22.4 to 13.4	.617
Dyspnoea	26.0	31.7	28.5	32.1	−2.5	−20.9 to 15.9	.784
Sleep disturbances	48.1	40.0	48.0	32.5	0.2	−19.6 to 19.9	.985
Appetite loss	20.4	32.6	18.7	28.9	1.7	−15.4 to 18.7	.845
Constipation	27.8	32.8	14.6	27.9	13.1	−3.6 to 29.8	.120
Diarrhoea	9.3	25.1	16.3	26.0	−7.0	−21.5 to 7.5	.339
Financial impact	29.6	37.7	20.3	31.5	9.3	−9.7 to 28.3	.330

Abbreviations: HCP, health care professional; PO, psycho‐oncological; QOL, quality of life.

## 4 CONCLUSION

Although several studies have shown the potential benefits of distress screening for need‐based POT,^26^, ^30^, ^35^ little is known about the effectiveness outside a study setting. Our investigation was designed to contribute knowledge on this aspect of PO screening.

More than twice as many patients were referred in the screening‐assisted referral period than during standard referral. The screening may have identified latent distress and POT wish that otherwise may have remained undetected. Additionally, completing the assessment may have reminded patients of issues they wished to discuss with a psycho‐oncologist. Finally, the standardised enquiry about patients' wish may have lowered the barrier to accepting help. The latter may have been especially true for patients with ThyCa, who have long been considered as experiencing only mild psychological distress because of their favourable prognosis.^20^, ^21^, ^22^, ^23^, ^36^

Patients with current or a history of depressive mood were most likely to express POT wish. As those patients were more likely to be or have been in psychosocial treatment or care, the barrier for them to accept professional support could have been lower. However, about half of patients with POT needs refused such treatment. Refusal of POT despite high levels of distress has previously been reported,^22^, ^30^ and destigmatisation of psychosocial problems could help in approaching patients in need.

We did not find significant differences in QLQ‐C30 scores between patients identified by standard referral vs screening‐assisted referral, but results must be interpreted with caution because of the small sample size and because of missing data, which was a result of the routine setting.

### 4.1 Limitations

The PO‐screening tool was based on a validated instrument. However, modifications were made to adapt it to clinical requirements. Hence, the applied cut‐off currently lacks validation. The strength of this tool is that it was developed in cooperation with clinical staff at the department where it is used. Therefore, it fits smoothly into the admission procedure and facilitates an early opportunity for patients to express their problems and accept POT if needed. Need‐based interprofessional implementation procedures are a prerequisite for the successful translation of developments from research to clinical practice.

Knowledge of patient pathways is important to gain insight into health service quality in clinical practice and may help to improve service planning or cost estimates. Therefore, a major strength of our approach is that we analysed real‐life data, which may provide an additional perspective on actual POT needs and wish. However, this real‐life setting meant that only half of the patients could be screened. This may partly be due to some patients refused participation in routine monitoring. Furthermore, the tight timeframe of daily clinical practice sometimes did not permit including patients in routine monitoring before they started radiopharmaceutical therapy. Psycho‐oncological screening is not useful for patients who have already started therapy, as psychological consultation cannot be performed during isolation. However, participants were more likely to be younger, female, and diagnosed with ThyCa. The proportion of patients with ThyCa at the department is far greater, and patients are usually diagnosed at a younger age being more familiar with electronic devises, which might have lowered the barrier to take part in the electronic assessments. Nevertheless, capacity problems and (electronic) screening scepticism must be encountered by, eg, educational work, staff training, or extension of resources. Nevertheless, HCPs play an important role in identifying distressed patients in need; therefore, PO screening should improve, not replace, this existing system. Further evidence for this is that 12 patients with negative PO screening were referred to POT by an HCP.^37^

An issue requiring special attention is that routine screening for distress entails some important ethical issues: The assurance of adequate treatment when screening represents a substantial psychosocial burden and that patients with noncancer‐related burden requiring professional attention are referred to experts within or outside the hospital. In identifying more patients who need POT, we might have to face new challenges regarding supply capacity and quality.^38^, ^39^, ^40^ An immediate link between screening and appropriate psychosocial support is an essential prerequisite for good clinical practice. Key elements that have been discussed in comparable implementation settings are HCP training and support (eg, a clear distribution of tasks); acceptability on both sides (for patients and HCPs); and a constant contiguity of screening results and treatment.^35^, ^39^ Hence, our next steps will be to focus on patients' and HCPs' general acceptance, ways to improve communication during the treatment process, and methods of lowering referral barriers to POT.^35^, ^39^ We assume that routine QOL and PO screening has the potential to change the way HCPs look after their patients' psychosocial issues. In addition, patients' self‐efficacy to monitor progression of mental and physical health and to self‐manage (disease‐related) issues must be addressed.^39^ Feedback of patients' own assessment results within the screening could be a first step.

### 4.2 Clinical implications

Routine PO screening may help to strengthen the HCPs' focus on psychosocial issues, reduce barriers to express the wish for POT, and may contribute to both referral pathways. However, long‐term follow‐up will show which benefits and challenges arise from this approach regarding effects on patients' well‐being or the quality of support.

## CONFLICT OF INTEREST

B.H. is a holder of part of the intellectual property rights of the Computer‐based Health Evaluation System software.

## ENDNOTES

*
Authors' translations without linguistic validation.
